# An epistatic interaction between pre-natal smoke exposure and socioeconomic status has a significant impact on bronchodilator drug response in African American youth with asthma

**DOI:** 10.1186/s13040-020-00218-7

**Published:** 2020-07-03

**Authors:** J. Magaña, M. G. Contreras, K. L. Keys, O. Risse-Adams, P. C. Goddard, A. M. Zeiger, A. C. Y. Mak, J. R. Elhawary, L. A. Samedy-Bates, E. Lee, N. Thakur, D. Hu, C. Eng, S. Salazar, S. Huntsman, T. Hu, E. G. Burchard, M. J. White

**Affiliations:** 1 Department of Medicine, University of California, 1550 4th Street, UCSF Rock Hall, Box 2911, San Francisco, CA 94158 USA; 2 Department of Biology, San Francisco State University, San Francisco, CA USA; 3 Berkeley Institute for Data Science, University of California, Berkeley, CA USA; 4 Lowell Science Research Program, Lowell High School, San Francisco, CA USA; 5 Department of Biology, University of California, Santa Cruz, CA USA; 6 Department of Genetics, Stanford University, Stanford, CA USA; 7 Sidney Kimmel Medical College, Thomas Jefferson University, Philadelphia, PA USA; 8 Department of Bioengineering and Therapeutic Sciences, University of California, San Francisco, CA USA; 9 School of Computing, Queen’s University, Kingston, ON Canada

**Keywords:** Epistatic interactions; Non-parametric methods; Asthma drug response; Health disparities; Pediatric asthma

## Abstract

**Background:**

Asthma is one of the leading chronic illnesses among children in the United States. Asthma prevalence is higher among African Americans (11.2%) compared to European Americans (7.7%). Bronchodilator medications are part of the first-line therapy, and the rescue medication, for acute asthma symptoms. Bronchodilator drug response (BDR) varies substantially among different racial/ethnic groups. Asthma prevalence in African Americans is only 3.5% higher than that of European Americans, however, asthma mortality among African Americans is four times that of European Americans; variation in BDR may play an important role in explaining this health disparity. To improve our understanding of disparate health outcomes in complex phenotypes such as BDR, it is important to consider interactions between environmental and biological variables.

**Results:**

We evaluated the impact of pairwise and three-variable interactions between environmental, social, and biological variables on BDR in 233 African American youth with asthma using Visualization of Statistical Epistasis Networks (ViSEN). ViSEN is a non-parametric entropy-based approach able to quantify interaction effects using an information-theory metric known as Information Gain (IG). We performed analyses in the full dataset and in sex-stratified subsets. Our analyses identified several interaction models significantly, and suggestively, associated with BDR. The strongest interaction significantly associated with BDR was a pairwise interaction between pre-natal smoke exposure and socioeconomic status (full dataset IG: 2.78%, *p* = 0.001; female IG: 7.27%, *p* = 0.004)). Sex-stratified analyses yielded divergent results for females and males, indicating the presence of sex-specific effects.

**Conclusions:**

Our study identified novel interaction effects significantly, and suggestively, associated with BDR in African American children with asthma. Notably, we found that all of the interactions identified by ViSEN were “pure” interaction effects, in that they were not the result of strong main effects on BDR, highlighting the complexity of the network of biological and environmental factors impacting this phenotype. Several associations uncovered by ViSEN would not have been detected using regression-based methods, thus emphasizing the importance of employing statistical methods optimized to detect both additive and non-additive interaction effects when studying complex phenotypes such as BDR. The information gained in this study increases our understanding and appreciation of the complex nature of the interactions between environmental and health-related factors that influence BDR and will be invaluable to biomedical researchers designing future studies.

## Background

Asthma is an inflammatory disease of the lower respiratory tract, characterized by symptomatic difficulty of breathing in affected individuals [1]. In the United States (U.S.), asthma is one of the leading chronic illnesses among children [2]. Asthma is also the most disparate common disease in pediatric populations, with asthma prevalence, morbidity, and mortality rates varying widely by racial/ethnic group [3]. Specifically, rates of asthma prevalence and mortality are two and four times higher, respectively, in African American children compared to European American children [3]. Measures of asthma morbidity, including emergency department visits and missed school days, are also higher in African American children compared to their European American counterparts [4]. Despite the higher asthma burden in the African American community, this population has been historically underrepresented in asthma research [5, 6]. Recent years have shown an increase in the inclusion of African Americans in large scale biomedical studies; however, this population is still comparatively understudied when contrasted with efforts aimed at European American populations [5, 6].

The disparity in asthma health outcomes across racial/ethnic groups may be due in part to a difference in drug response. Bronchodilators, specifically short acting β_2_-agonist medications such as albuterol, are the most commonly prescribed asthma medication in the United States [7, 8]. Bronchodilator drug response (BDR) is the amount of airway obstruction that is reversible after the administration of bronchodilator medication. BDR varies significantly between racial/ethnic groups [9–11]. Alarmingly, compared to other racial/ethnic groups, African American children with moderate-to-severe asthma respond poorly to bronchodilators, ranking second worst among all demographic groups [11].

The estimated genetic heritability of bronchodilator drug response is approximately 28.5% [12]. However, this estimate only represents the additive effect of each genetic factor on BDR variability and does not account for gene/bio-environment, gene-gene, or variant-variant effects.

For example, while measures of air pollution, socioeconomic status, genetic ancestry, and obesity have all been independently associated with BDR and/or other asthma-related phenotypes, the amount of variation in BDR independently explained by each of these variables is relatively small, leaving a large portion of the variation in BDR unexplained [7, 13–16]. A portion of the undefined variation in BDR is likely explained by gene-gene, gene-environment, or biology-environment (bio-environment) interactions [17]. While these interactions could be additive or non-additive in nature, historically research in complex phenotypes, such as BDR, has traditionally focused primarily on the identification of additive interaction effects through the use of regression-based methods. Synergistic non-additive interactions (epistatic interactions) have recently been recognized as a significant source of variation underlying complex diseases [18–20]. The widely employed regression-based models characteristic of large-scale genetic/biomedical/epidemiological studies of complex disease, while adept at detecting additive, or additive, interaction effects, are not well powered to detect non-additive interactions. In addition, the majority of studies investigating interaction effects in complex diseases have used additive models in largely European populations [21–23]. Consequently, there is a significant lack of research investigating epistatic interaction effects between environmental, psychosocial, demographic, and clinical factors in asthma research. Furthermore, the lack of biomedical research in non-European populations perpetuates asthma health disparities, especially for those populations that carry a high disease burden, such as African Americans.

Visualization of Statistical Epistasis Networks (ViSEN) is a statistical program that optimizes detection of epistatic interactions through information-theoretic quantities, and is able to include multiple types of data as discrete random variables [20, 24]. ViSEN is also able to identify and quantify both additive and non-additive interactions and provide intuitive visualization of the potentially complex relationships between large numbers of variables using a network-based approach. Additionally, ViSEN has been shown to be more powerful than standard regression-based methods in detecting interaction effects, suggesting that ViSEN is a promising investigative tool that can be applied to studies of complex traits such as BDR [20, 24–28].

In this study, we conducted pairwise (two-variable) and higher order (three-variable) interaction analyses using ViSEN to study the impact of both additive and epistatic interactions between social, psychological, and biological variables on BDR in 233 African American youth with asthma. We then performed these analyses in sex-stratified subsets to identify sex-specific effects in our study population. Our study is the first to rigorously interrogate clinical, environmental, and demographic information to identify hidden non-additive interactions affecting BDR in African American children with asthma. By identifying previously overlooked epistatic interactions that significantly influence BDR in African American youth with asthma; our study aims to provide novel information that can aid in characterizing targets for future health intervention strategies and improving the design of future studies of BDR.

## Results

### Study participants

The SAGE study included African American participants aged 8 to 21 years with and without asthma. After excluding Individuals without asthma and individuals missing any phenotype data, a subset of 617 SAGE participants remained for potential inclusion in our study. After applying an additional quality control procedure, local case control (LCC) subsampling (see Methods section), to remove the potentially confounding effect of age, 233 individuals with asthma remained for study inclusion (Males = 136, Females = 97; see Additional File 1). In the full dataset, there were no significant associations between individual predictor variables and BDR status identified by either ViSEN or standard descriptive statistics (Table 1). Similarly, we explored the possibility of main effects in sex-stratified subsets of our population. For males, no main effects were identified, while in females, ViSEN identified an association between an experience of discrimination (EOD) and BDR responder status (*p* = 0.01); however, this association was not significant after correction for multiple testing (Additional File 2, Additional File 3). A borderline association between SES and BDR status (*p* = 0.05) was identified by both ViSEN and standard descriptive statistics, however, this association was not significant after correction for multiple testing (Supplemental Table 2, Supplemental Table 3) (Additional File 2, Additional File 3).

**Table 1:** Study Participant Demographics

				ViSEN	Descriptive Statistics
Categorical Variable		BDR Responders	BDR Non-Responders	*p*-value^1^	*p*-value^2^
Sample Size, N		118	115	–	–
Sex (% Female)		42%	42%	1.00	1.00
Age, yrs. (Mean, [SE])		(14, [0.346])	(14, [0.323])	0.67	0.37^3^
Body Mass Index	Obese	47	39	0.44	0.42
	Non-Obese	71	76		
Experience of Discrimination	Yes	65	49	0.06	0.08
	No	53	66		
Pre-natal Smoke Exposure	Yes	21	19	0.87	0.93
	No	97	96		
Socioeconomic Status	> Low	81	73	0.41	0.49
	Low	37	42		
Air Pollution (NO_2_)	≥ Median	62	54	0.43	0.47
	< Median	56	61		
Global African Ancestry	≥ 80%	82	67	0.08	0.10
	< 80%	36	48		

Summary statistics for all phenotypic data included for analysis in this study are presented above. The Bonferroni method was used to correct for multiple testing (threshold for statistical significance: permutation unadj. *p* ≤ 0.006 (0.05/8 tests)). P-values represent the significance of the main effects, of specified variables on BDR responder status. ^1^p-values calculated from ViSEN’s Mutual Information (MI) Test. MI is a metric that quantifies the reduction in uncertainty about the distribution of one variable given an understanding of the other; ^2^p-values calculated from the χ^2^ Test of Independence unless otherwise indicated; ^3^p-values calculated from Wilcoxon Rank Sum test

### ViSEN pairwise (two variable) interaction effects

**Fig. 1:**
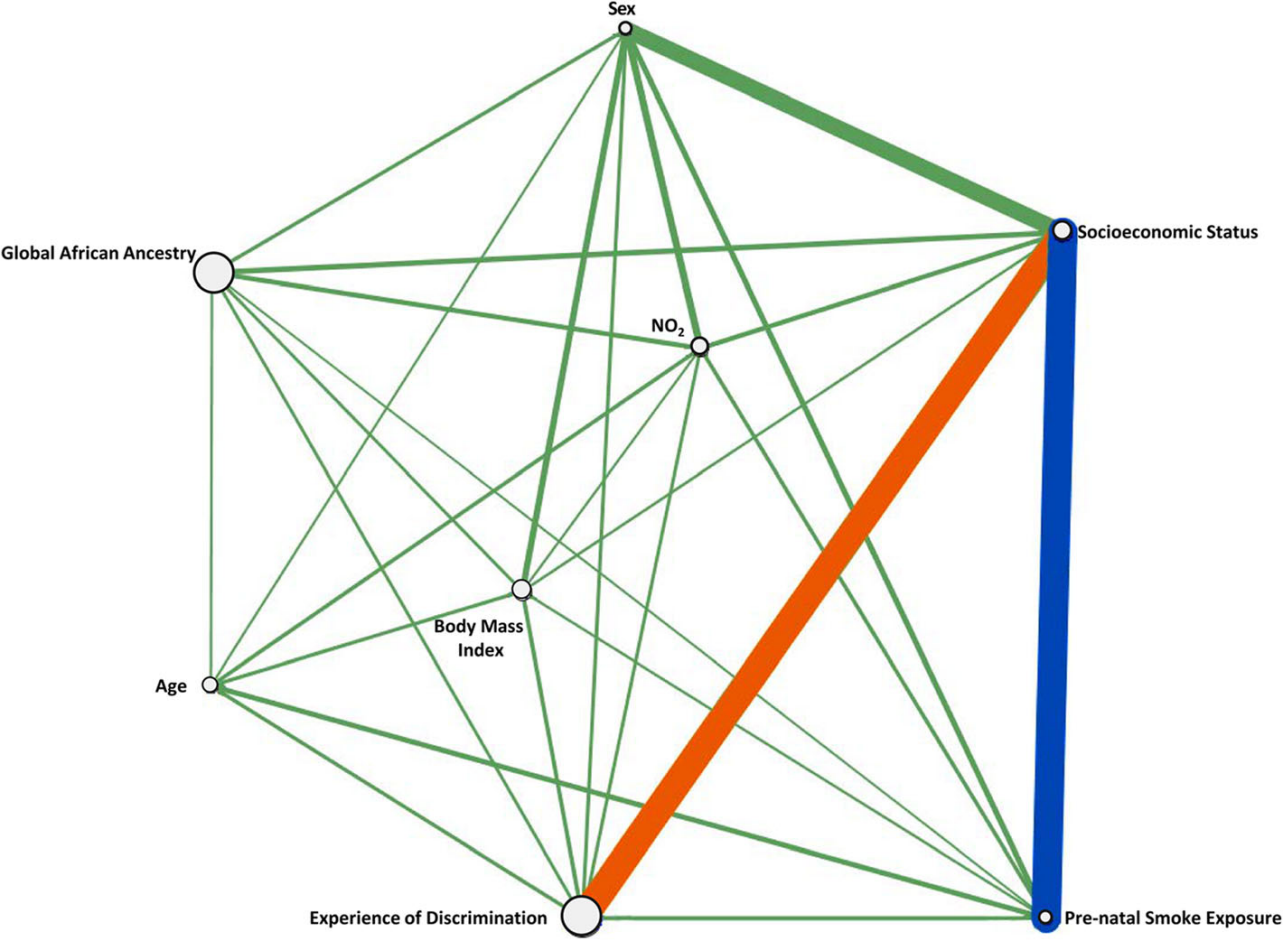
Visualization of Pairwise Interactions in the Full Dataset. Visual representation of the interaction network for pairwise models in the full dataset. The size of an individual node corresponds to the amount of Mutual Information (MI) resulting from the main effects of each variable. The strength of significant pairwise interactions corresponds to the thickness of the lines connecting individual nodes along the network. Line color denotes statistical significance of pairwise interactions; blue lines represent interactions significantly associated with BDR after correction for multiple testing, (Bonferroni significance threshold *p* < 0.002) orange lines represent interactions suggestively associated with BDR after correction for multiple testing (Bonferroni suggestive threshold *p* < 0.004)

We identified one pairwise interaction significantly associated with BDR (significance threshold, IG *p* ≤ 0.001) in the full dataset using ViSEN, and one additional interaction that was suggestively associated (suggestive threshold, IG permutation unadj. *p* ≤ 0.004) with BDR (Table 2A, Fig. 1). In order of strength (IG) and significance, our ViSEN identified interactions were: [1] pre-natal smoke exposure and socioeconomic status (PSE x SES) (IG = 2.78%, permutation unadj. *p* = 0.001) and [2] experience of discrimination and socioeconomic status (EOD x SES) (IG = 2.71%, permutation unadj. *p* = 0.002) (Table 2A and Fig. 1). Logistic regression analysis was unable to produce any significantly associated models; however, our significant and suggestive models were identified at the suggestive level of significance in logistic regression (Table 2A). An association between sex and socioeconomic status (Sex x SES; IG = 1.47%, permutation unadj. *p* = 0.026) identified by ViSEN, though not significantly or suggestively associated with BDR after correction for multiple testing, indicated the potential presence of sex-specific effects in our dataset. Socioeconomic status (SES) appeared in each associated interaction model identified by ViSEN, however, this variable did not show any significant main effects (Mutual Information *p*-value = 0.49, χ^2^*p*-value = 0.41; Table 1).

**Table 2:** Age Adjusted Pairwise Interaction Models Associated with BDR Identified by ViSEN

**A. Full Dataset**						
			**ViSEN Analysis**		**Logistic Regression**	
**Variable 1**		**Variable 2**	**IG**	***p*-value**	**OR**	***p*-value**
Pre-natal Smoke Exposure		Socioeconomic Status	**2.78%**	**0.001**	0.105	0.004^a^
Experience of Discrimination		Socioeconomic Status	2.71%	0.002^a^	0.166	0.003^a^
Sex		Socioeconomic Status	1.47%	0.026	0.291	0.031
**B. Sex-Stratified Subsets**						
			**ViSEN Analysis**		**Logistic Regression**	
**Subset**	**Variable 1**	**Variable 2**	**IG**	***p*-value**	**OR**	***p*-value**
Female *N* = 97	Pre-natal Smoke Exposure	Socioeconomic Status	7.27%	0.004^a^	0.054	0.033
	Experience of Discrimination	Socioeconomic Status	4.52%	0.016	0.093	0.014
Male *N* = 136	Experience of Discrimination	Socioeconomic Status	3.04%	0.015	0.135	0.035
	Pre-natal Smoke Exposure	Socioeconomic Status	2.69%	0.032	0.082	0.038

Information Gain (IG) and unadjusted permutation *p-value* results for select interaction models (permutation unadj. *p* < 0.05) with BDR identified by the age adjusted ViSEN and logistic regression analyses. Positive IG values indicate synergistic interactions, negative IG values indicate redundant models. The Bonferroni method was used to correct for multiple testing. Bonferroni familywise error rate (FWER) thresholds of 0.05 and 0.1 were used to determine significantly associated models (permutation unadj. *p* ≤ 0.002) and suggestively associated (permutation unadj. *p* ≤ 0.004), respectively. Models significantly associated with BDR after correction for multiple testing are highlighted in **BOLD**. Models suggestively associated with BDR after correction for multiple testing are indicated with ^a^. Logistic regression models were adjusted for age and the marginal effects of each independent variable included in the specified interaction model. SES: Socioeconomic Status; Smoking: Pre-natal smoke exposure; EOD: Experience of Discrimination; OR: Odd’s Ratio

ViSEN analysis of pairwise interactions performed in sex-stratified subsets of our study population also identified the PSE x SES interaction as the strongest effect in our study (Table 2B). In females, the association remained suggestively associated with BDR after correction for multiple testing (IG = 7.27%, permutation unadj. *p* = 0.004) (Table 2B). After correction for multiple testing, logistic regression was unable to reliably detect the PSE x SES interaction effect on BDR (*p*-value = 0.033). ViSEN analysis in the male-only subset revealed that the PSE x SES interaction was once again the strongest pairwise effect, however, in males the association was not significant after correction for multiple testing (IG = 3.04%, permutation unadj. *p* = 0.015) (Table 2B).

**Fig. 2:**
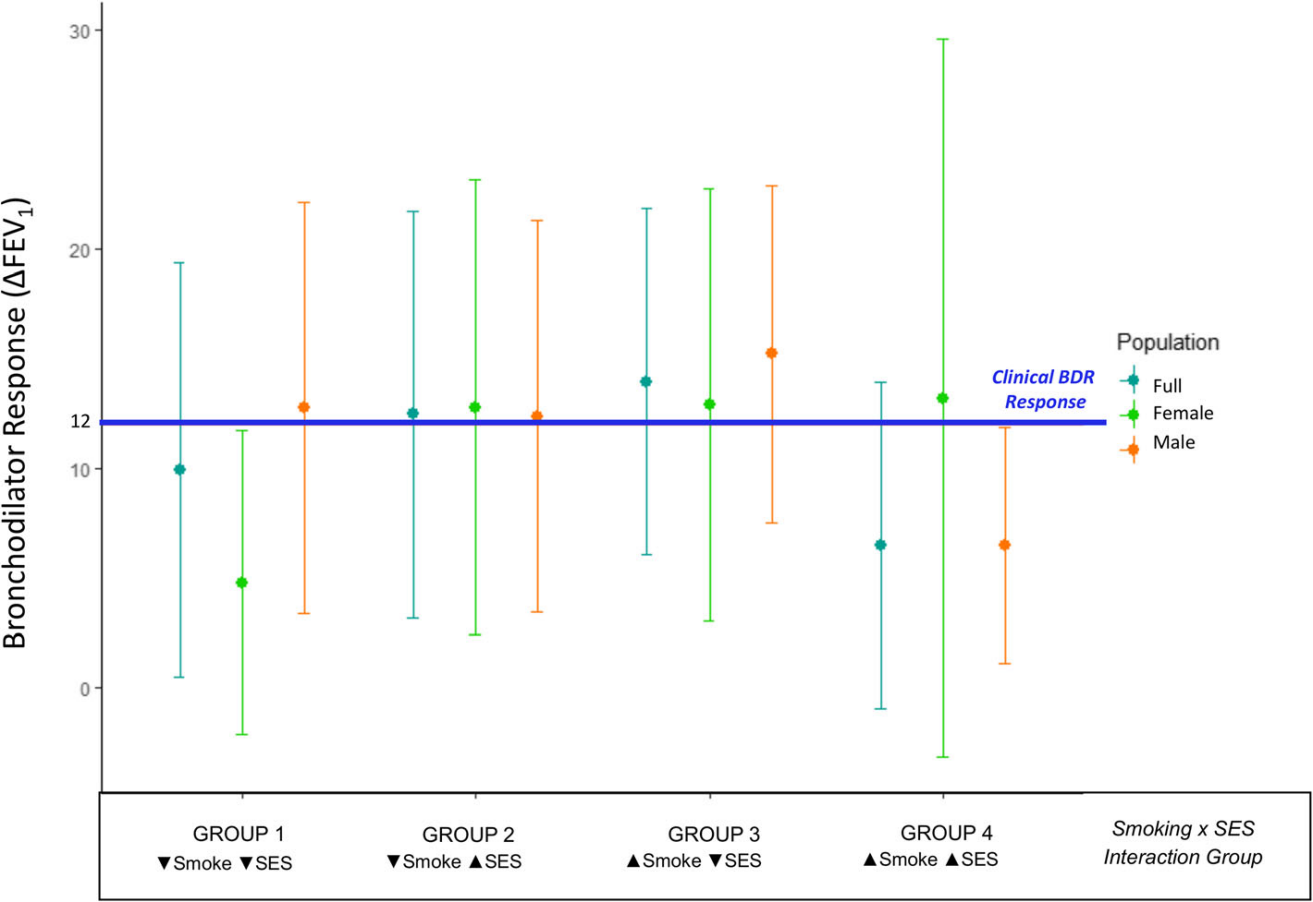
Distribution of Bronchodilator Drug Response by Pre-natal Smoke Exposure and Socioeconomic Status (Smoke x SES) Interaction Group. Grouped Error Plot Graph of Bronchodilator Drug Response by Smoke x SES interaction group membership. Vertical lines denote the interquartile range (IQR) of group data; circles indicate the group median. Line color indicates dataset (full, male-only, female-only). Blue horizontal line indicates threshold for clinical response to bronchodilator treatment (∆ FEV_1_ ≥ 12%). ∆ FEV_1_ is the difference in % of Predicted FEV_1_ achieved before and after bronchodilator treatment. ▼ means that the group median < study population median; ▲ means that the group median ≥ study population median. Smoke denotes pre-natal smoke exposure, while SES indicates socioeconomic status

**Fig. 3:**
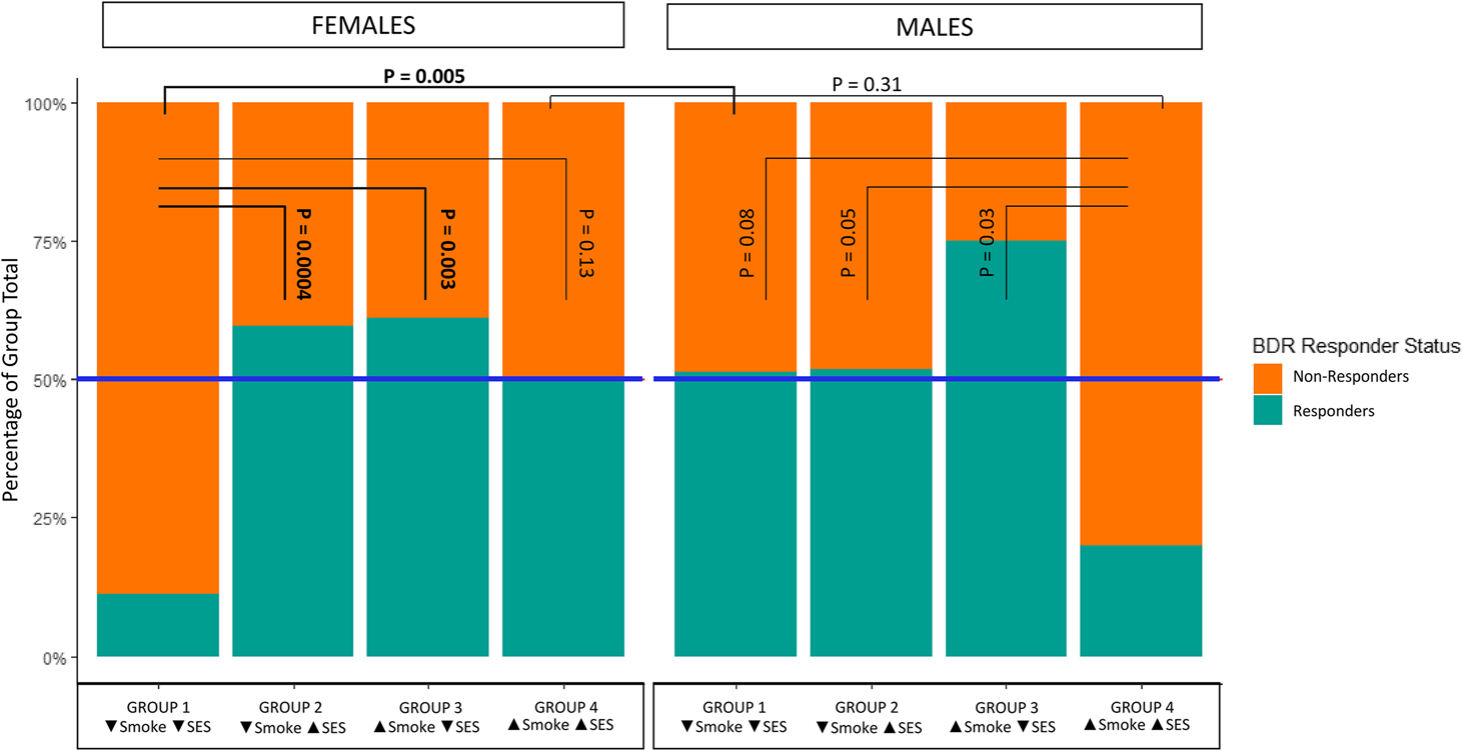
Proportion of Bronchodilator Drug Responders and Non-Responders by Pre-natal Smoke Exposure and Socioeconomic Status (Smoke x SES) Interaction Group. Stacked Box Plots of Proportion of BDR responders and non-responders by Smoke x SES Interaction group membership separated by sex. Blue horizontal line indicates 50%. Bars are colored by BDR responder status. *P*-values presented are from the χ^2^ statistic of the two-group test of proportions; when bin sizes were < 5, the Fisher’s Exact test was used instead. P-values in **bold** font were significant after Bonferroni correction for multiple testing

The PSE x SES interaction was the only interaction model that was significantly associated in the full dataset, and suggestively or marginally associated in both of our sex-stratified analyses (Table 2A, B). As the strength, if not the significance, of this interaction appeared to be conserved across all three populations, we performed post-hoc analyses and data visualization to better characterize the nature of the relationship between the PSE x SES interaction and BDR (Figs. 2 and 3) (Supplemental Figure 1; Additional File 5). Participants were separated into groups defined by their PSE x SES interaction phenotype as follows: [1] no PSE and low SES, [2] no PSE and med/high SES, [3] PSE and low SES, and [4] PSE and med/high SES. Data visualization revealed that the distribution of continuous BDR measurements varied by sex (Fig. 2). By visualizing the interquartile range (IQR) and median of each interaction group in Fig. 2, we were able to determine that the majority of females in Group 1 were beneath the critical threshold for BDR clinical response (∆ FEV1 ≥ 12%)(Fig. 2); a similar phenomenon was seen in Group 4 males where the majority of the Groups BDR distribution was beneath the clinical threshold for response (Fig. 2). Further analysis revealed that, in females, Group 1 (no PSE and low SES) had a significantly lower proportion of BDR responders compared to all other groups in females with the exception of Group 4 (Fig. 3). Group 1 in females also had a significantly lower proportion of BDR responders than Group 1 in males (*p*-value = 0.005) (Fig. 3). In males, Group 4 (PSE and med/high SES) had the lowest proportion of BDR responders compared to all other interaction groups in males (Fig. 3). Pairwise testing revealed that the difference in proportion of BDR responders between Group 4 in males and Groups 1–3 in males was not statistically significant after correction for multiple testing (Fig. 3). While proportion of BDR responders in Group 4 in males was also lower than the corresponding Group proportion in females, this difference was also not statistically significant (Fig. 3).

### ViSEN higher-order (three-variable) interaction effects

We identified five higher-order (three-variable) interactions associated with BDR in the full dataset using ViSEN. After correction for multiple testing, only one interaction model containing an experience of discrimination, age, and SES (EOD x AGE x SES) remained suggestively associated with BDR (IG = 2.39%, permutation unadj. *p* = 0.003) (Table 3A). Logistic regression analysis identified three of the five marginally associated interactions, but after correction for multiple testing, none of the logistic regression results were significantly or suggestively associated with BDR. Additionally, one of the interaction models (an experience of discrimination, pre-natal smoke exposure, NO_2_ air pollution exposure; EOD x PSE x NO2) was unable to be reliably evaluated using logistic regression due to limitations common to regression-based analyses in small sample sizes (i.e. separation and multicollinearity) (Table 3A).

**Table 3:** Age Adjusted Higher-Order Interaction Models Associated with BDR identified by ViSEN

**A. Full Dataset**							
				**ViSEN Analysis**		**Logistic** **Regression**	
				**Age Adjusted**			
**Variable 1**		**Variable 2**	**Variable 3**	**IG**	***p*-value**	**OR**	***p*-value**
Experience of Discrimination		Age	Socioeconomic Status	2.39%	0.003	0.029	0.008
Experience of Discrimination		African Ancestry	Socioeconomic Status	1.71%	0.012	0.026	0.040
Experience of Discrimination		NO_2_ Air Pollution	Socioeconomic Status	1.80%	0.017	0.043	0.013
Sex		Pre-natal Smoke Exposure	Socioeconomic Status	1.82%	0.041	1.525	0.817
Experience of Discrimination		Pre-natal Smoke Exposure	NO_2_ Air Pollution	1.66%	0.043	–	–
**B. Sex-Stratified Subsets**							
				**ViSEN Analysis**		**Logistic** **Regression**	
				**Age Adjusted**			
**Subset**	**Variable 1**	**Variable 2**	**Variable 3**	**IG**	***p*-value**	**OR**	***p*-value**
Female	Experience of Discrimination	Pre-natal Smoke Exposure	African Ancestry	2.73%	0.018	1.000	1.000
	Pre-natal Smoke Exposure	Socioeconomic Status	NO_2_ Air Pollution	4.92%	0.030	0.091	0.413
	Experience of Discrimination	Pre-natal Smoke Exposure	Age	4.01%	0.039	–	–
Male	Experience of Discrimination	African Ancestry	Socioeconomic Status	5.11%	0.001^a^	0.004	0.005
	Experience of Discrimination	NO_2_ Air Pollution	Socioeconomic Status	3.95%	0.008	0.012	0.015
	Experience of Discrimination	Age	Socioeconomic Status	3.90%	0.012	0.012	0.017
	Experience of Discrimination	African Ancestry	NO_2_ Air Pollution	2.44%	0.038	–	–

Information Gain (IG) and unadjusted permutation *p-value* results for select interaction models associated (permutation unadj. p < 0.05) with BDR identified by the age adjusted ViSEN and logistic regression analyses. Positive IG values indicate synergistic interactions, negative IG values indicate redundant models. The Bonferroni method was used to correct for multiple testing. Bonferroni familywise error rate (FWER) thresholds of 0.05 and 0.1 were used to determine significantly associated models (permutation unadj. *p* ≤ 0.0009) and suggestively associated (permutation unadj. p ≤ 0.002), respectively. Models significantly associated with BDR after correction for multiple testing are highlighted in **BOLD**. Models suggestively associated with BDR after correction for multiple testing are indicated with ^a^. Logistic regression analysis was performed in the same LCC age adjusted dataset as VISEN analyses for accurate comparison of results; to maintain consistency with ViSEN analyses regression models were adjusted for the marginal effects of each independent variable included in the specified interaction model. When interaction bin size was < 5, Firth’s Bias-Reduced logistic regression OR and *p*-values are presented. ---: model could not be accurately assessed by regression modeling due to deviation from model assumptions of no collinearity and no complete or quasi-complete separation; OR: Odd’s Ratio

ViSEN analysis of three-variable interactions performed in sex-stratified subsets of our study population generated discordant results (Table 3B). Although several marginally associated interactions were identified in both sexes, there was no overlap in identified models between the two sub-groups. In terms of model composition, models identified in females all contained pre-natal smoke exposure while models identified in the male-only sub-sample all contained an experience of discrimination (EOD). After correction for multiple testing, a single interaction model in males composed of EOD, African ancestry, and socioeconomic status was marginally significant (IG = 5.11%, permutation unadj. *p* = 0.001) (Table 3B). Logistic regression analysis was unable to reliably detect any of the ViSEN-identified three-variable interaction effects; notably, three models were unable to be assessed by logistic regression at all due to deviation from model parameters (model testing revealed small bin sizes, quasi-separation, and/or multicollinearity).

## Discussion

The high inter-individual variability of BDR between racial/ethnic populations may contribute to disparities in asthma morbidity and mortality observed in African American children with asthma. An existing pharmacogenomic study has characterized genetic components which may explain response to albuterol in this population [29]. Related studies have also noted ethnic-specific and other phenotypic differences in bronchodilator drug responsiveness [9–11]. However, few have observed the joint effect of biological (including genetic ancestry estimates) and environmental variables influencing BDR. Investigating epistatic interactions is important to understanding variation in BDR in the context of asthma outcomes, such as morbidity and mortality, and improving health equity across the U.S. Social determinants of health are recognized as some the most predictive factors contributing to individual health outcomes. Therefore, the inclusion of variables describing our “built environment” (i.e. psychosocial factors such as socioeconomic status, an experience of discrimination, etc.) in population-based studies of BDR is crucial.

It is also important to understand that parametric methods, such as logistic regression, are the statistical tool of choice in most scientific efforts to characterize interactions associated with BDR and other complex biological phenotypes. The strength of using parametric methods is that they are well-understood and therefore generally interpretable. However, some parametric methods make data assumptions (normality of variable sample distributions, patterns of correlation between variables, and/or the distributional relationship of any identified interactions to name a few) that may not hold true for many complex traits. To the best of our knowledge, our study is the first to demonstrate an integrative, investigative approach, which analyzes the interaction between clinical, biological, environmental, and psychosocial factors affecting drug response in African American children and adolescents using a non-parametric method optimized to detect epistatic interactions.

In our study, ViSEN consistently identified novel pairwise and higher-order interactions occurring within our study population that, to our knowledge, have not been discussed elsewhere. Notably, the most informative interaction (IG = 7.27%) occurring in females between pre-natal smoke exposure and socioeconomic status (PSE x SES) was discovered completely independent of strong main effects for either variable (PSE Mutual Information permutation unadj. *p* = 0.87; SES Mutual Information permutation unadj. *p* = 0.41) (Table 1). Our sex-stratified analyses revealed that this interaction had a complex relationship with BDR, with interaction group membership correlating with different effects on BDR for males and females (Figs. 2 and 3). This model was not identified as significantly associating with BDR using logistic regression. The strongest higher order interaction model (EOD x AFR x SES) was also not identified as significantly associating with BDR by logistic regression analysis. Importantly, of the eight significant interaction effects identified by ViSEN in the full dataset, none of these effects were detected using logistic regression (*p*-values not significant after correction for multiple testing). These results suggest that the majority of the interaction effects revealed by ViSEN are synergistic (non-additive) in nature and therefore may not always be reliably identified, particularly in smaller datasets, using standard regression-based methods. Our results also support the theory presented by Hu et al. that ViSEN is better powered to detect interaction effects under certain conditions (i.e. no main effects or weak main effects) [20, 30].

Another interesting result of our study was the discordance in the relationship between interaction group membership for our significant interaction (PSE x SES) and BDR seen between male- and female-only subsets. Our results suggest the presence of significant sex-specific differences in the interactions affecting BDR status, especially in our more complex higher-order models. Also, while similar pairwise interactions were detected in both males and females, our post-hoc visualization efforts have shown the effect of PSE x SES interaction group membership to be significantly different between the disparate sexes. Another important revelation of our study was the ubiquity of higher-order interaction effects on BDR. In both the full dataset and sex-stratified subsets, ViSEN detected more three-variable interaction models associated with BDR compared to pairwise interaction models (Tables 2 and 3). This suggests that incorporating the study of more complex models may lead to novel discoveries in this complex phenotype.

Interpreting results from ViSEN entails several considerations. The first consideration involves the nature of the variables and their method of collection. BDR represents a clinical continuous measurement obtained via spirometry, but other variables such as an experience of discrimination were collected via a self-reported questionnaire. Therefore, measurement error may have contributed to the detection of interactions within this study, especially in variables that are not easily validated by repeated measurements. However, in our study we have rigorously identified classifications for each variable and validated our measurements by either consulting clinical guidelines or referencing previous literature [13, 14, 31–34]. All phenotype data included in this study has also been successfully used in other studies relating to asthma phenotypes [13, 14, 31–34]. Again, it is paramount to consider that regardless of the rigor of data collection, ViSEN must collapse continuous variables into a ranked form and therefore some information will be lost as result. It should also be noted that dichotomization of continuous predictor variables can lead to over-simplification of the resulting interaction models. A countermeasure to this is the use of informative clinically or biologically relevant thresholds for dichotomization; while this will not entirely counteract simplification of the underlying interaction network, it may potentially increase the interpretability of the resulting interaction models.

Furthermore, the need for replication and validation of our results should not be understated. The comparatively limited availability of BDR data in African American children with asthma due to historic underrepresentation of minority populations in biomedical studies, coupled with the decrease in sample size following the LCC subsampling method used to correct for confounders in ViSEN analysis, served to substantially decrease power in our analyses. While we contend that our results support the utility of ViSEN to identify novel interactions, it is possible that some interactions went undiscovered, or may not have passed correction for multiple testing, due to the modest sample size of our study population. It should also be noted that because our results have yet to be replicated in diverse populations, it is unclear as to whether the interactions identified in our study are cosmopolitan or population-specific. We recognized that replication of our results may be difficult, even in other African-descent populations, as differences in environmental exposure frequencies and biological predictors (obesity, genetic ancestry, etc.) will vary both within and between groups. In an attempt to counteract this, we were cognizant to standardize our phenotypic variables where possible, to emphasize clinical importance during the ranking of data and to apply reproducible methodology. We aim to continue this work in other underrepresented populations for whom we have available data.

While ViSEN can identify additive and non-additive interactions and quantify the amount of information provided by these models, it does not provide the directionality of these effects (i.e. whether membership in different interaction group correlates positively/negatively to BDR responsiveness). To mitigate this limitation for studies in which information on directionality of identified interaction effects would be useful, ViSEN can be supplemented with additional visualization techniques and pairwise tests, such as the Dunn test or the two group test of proportions (as demonstrated in our study) [35, 36]. However, it should be noted that while supplementation of ViSEN analysis in this way is possible, results from these post-hoc tests may not be easily interpretable for every phenotype or interaction model. It will be the responsibility of individual researchers to determine if their specific study lends itself to this type of further analyses. Another potential limitation of the ViSEN method is that the program does not include a direct method to adjust for potentially confounding variables. These effects must be adjusted for prior to ViSEN analyses using an appropriate method; for the current study, the LCC approach was used to adjust for age as a potentially confounding variable. It is important to note that the effects of a potential confounder must be removed from both the outcome variable, and any predictor variable that is also significantly affected by the confounder. Consequently, to ensure correct inference of ViSEN results, we stress the need to thoroughly investigate the relationship between the outcome and predictor(s) to ensure that confounding effects have been appropriately controlled. For instances in which researchers require outcomes and covariates to remain continuous, ViSEN can potentially be used as a filtering analysis. For example, quantitative multifactor dimensionality reduction (qMDR) is a non-parametric approach that is able to handle continuous outcomes directly and continuous covariates indirectly [37]. Specifically, qMDR can adjust for the effects of continuous covariates using the “regress out” method in which covariates are regressed on a continuous outcome and the resulting residuals are used as the covariate-corrected input for qMDR analysis. QMDR is not able to distinguish “pure” interactions from those that are simply the result of strong main effects. Interaction analysis could be run in both qMDR (with continuous outcome) and ViSEN (with dichotomous outcome); concordant results would suggest “true” interactions.

## Conclusion

We identified novel interaction models significantly impacting BDR in a population that carries a high disease burden (increased asthma morbidity and mortality compared to European American children with asthma) and has been historically understudied and underserved. We contend that a significant portion of interactions between biological and environmental affecting complex phenotypes like BDR are non-additive in nature, and entropy-based methods, such as ViSEN, may be better able to reliably detect these effects under certain conditions, as the results of this study indicate. The diversity and complexity of the interactions impacting BDR should be embraced by incorporating non-parametric methods, such as ViSEN, into biomedical research aimed at exploring complex traits.

The nonparametric nature of ViSEN facilitates analyses that are more inclusive of the varying relationships and interactions between health factors likely impacting complex clinical phenotypes versus traditional regression models as we highlight in this study of BDR. Our study represents a collaboration of computer science, biology, and epidemiology that lead to the identification of a novel interaction between pre-natal smoke exposure and socioeconomic status that was significantly associated with BDR in African American children with asthma. We believe that further collaboration between these fields is necessary to conduct comprehensive studies of BDR that will lead to continued novel discoveries and improved understanding of the etiology of BDR.

## Methods

### Study population

The Study of African Americans, Asthma, Genes, & Environments (SAGE) is a case-control study consisting of 1710 participants ranging from ages 8 to 21 years old recruited from the San Francisco Bay Area between 2008 and 2014. The SAGE study protocol and patient population have been previously described in further detail elsewhere [14, 29, 31]. Briefly, all SAGE participants included in this study self-identified as African American, as did their parents and all four grandparents. All participants presented no history of other lung or chronic non-allergic illnesses upon study enrollment. Trained interviewers administered questionnaires to the participants and/or the parents/caretakers of the participants to collect basic demographic information, medical histories, and environmental exposure-related information [14].

A total of 617 participants with physician-diagnosed asthma from SAGE, with complete data on sex, age, global African genetic ancestry, body mass index (BMI), any experience of discrimination, socioeconomic status, pre-natal smoke exposure, ambient NO_2_ exposure over the first year of life, and BDR were available for potential inclusion in our study (Additional File 1). After a final data processing procedure (see Methods section, “Application of ViSEN in the Current Study”), a total of 233 participants remained for inclusion in our study (Supplemental Table 1) (Additional File 1). Our sex-stratified subsets consisted of 136 male and 97 female individuals (Supplemental Table 2, Supplemental Table 3) (Additional File 2 and Additional File 3). Appropriate descriptive statistics for participants in the full dataset, as well as sex-stratified subsets, were generated using the R statistical computing environment (Table 1, Supplemental Table 2, Supplemental Table 3) (Additional File 2, and Additional File 3). ViSEN does not accommodate continuous variables. Consequently, the outcome variable and all explanatory variables included in interaction analyses were dichotomized prior to analysis as described below and in Additional File 4.

### Bronchodilator drug response (BDR)

The primary outcome of this study is bronchodilator responder status. Responder status was determined from individual spirometry measurements taken before and after administration of albuterol. Following American Thoracic Society recommendations, pulmonary function was measured prior to albuterol administration and then repeated 15 min after administration of four puffs (90 μg/puff) of albuterol [38]. This process was repeated a third time after a second dosage of albuterol: two puffs for participants under the age of 16 and four puffs for older participants [29]. Asthma medications were withheld from participants 12 h before spirometry [39]. BDR (ΔFEV_1_) was calculated as the mean percentage change in measured Forced Expiratory Volume (FEV_1_) measured before (pre-FEV_1_) and after (post-FEV_1_) albuterol administration, using the post-albuterol spirometry with the maximal change ((post-FEV_1_ – pre-FEV_1_) / pre-FEV_1_) × 100%. For each participant in this study, BDR (ΔFEV_1_) was used to classify bronchodilator responder status as either a responder, ≥ 12%, or a non-responder, < 12% [34]. We excluded two participants who were statistical outliers for BDR (raw values) as previously described [40].

### Age

The participant’s age was calculated as the difference between the age of enrollment (the date on the eligibility form) and the date of birth. Discrete variables ranked from 0 to 1 were generated depending on whether an individual was aged below (ranked 0), or at and above the median for the population (ranked 1).

### Global African ancestry

Participants included in this study were previously genotyped using the Axiom® LAT1 array (World Array 4, Affymetrix, Santa Clara, CA) [41, 42]. For every individual, we estimated the genetic proportion contributed by an African ancestral population. These estimates, obtained using an unsupervised run of ADMIXTURE, were considered as an average over each individual’s entire genome to comprise a global ancestry variable [43]. Reference haplotypes of African and European individuals used in ADMIXTURE were gathered from the HapMap phase III YRI and CEU populations [15]. In this study, values for global African ancestry were dichotomized according to their distribution above or equal to (1) or below (0) the U.S. national average of 80% global African ancestry [44].

### Body mass index

At the time of enrollment, each study participant was measured via a calibrated scale and stadiometer for weight (kg) and height (m), respectively. Body mass index percentile values were subsequently calculated through the following formula: BMI = (kg)/ (m^2^). BMI percentile values were generated using guidelines for BMI categories from the U.S. Centers for Disease and Control and Prevention Growth Charts. BMI percentile values were dichotomized as either 0 or 1 depending on whether they fell below (< 95%) or above/equal to (≥ 95%) the Obese BMI classification [16].

### Perceived experience of discrimination

Self-reported racial/ethnic discrimination was ascertained using the Experiences of Discrimination Questionnaire [45]. Consistent with previous studies, we included questions pertaining to our population: “Have you ever experienced discrimination, been prevented from doing something, or been hassled or made to feel inferior, in any of the following situations because of your race, ethnicity, color, or language? (1) At School; (2) Getting medical care; (3) Getting services in a store or restaurant; and (4) On the street or in a public setting”; with choice for each question of *Yes* or *No*. [46, 47] Experiences of discrimination were dichotomized as none or any (affirmative answer to at least one situation). Interviewers required permission of caretakers to administer questions to participants equal to or less than 16 years of age. Perceived experiences of discrimination were reported at time of recruitment [47].

### Pre-natal smoking

Pre-natal exposure to smoke was determined from questionnaire information regarding the self-reported smoking status of participant’s mother during pregnancy. Binary values were assigned for smoking status based on whether the mother was a non-smoker (0) or active smoker (1) during the pregnancy of the participant.

### Socioeconomic status

We created a composite index for socioeconomic status (SES) derived from three socioeconomic indicators: mother’s educational attainment, insurance status, and household income as previously described [32]. Each component variable was independently assigned a value scored on a three-point scale ranging from low SES (0), to medium SES (1), to high SES (2). Finally, for the purpose of our study, individuals were classified as either having a low (0) or medium/high (1) composite socioeconomic scores.

### Nitrogen dioxide exposure

TomTom/Tele Atlas EZ-Locate software (TomTom, Amsterdam, The Netherlands) was used to assign geographic coordinates for each participant’s residential history. We collected regional ambient air pollution data from the US Environmental Protection Agency Air Quality System based on these geographic coordinates [13]. Measures of average ambient NO_2_ exposure (μg/ppb) were estimated over the first year of each participant’s life. If the participant moved during this period, NO_2_ exposure was weighted depending on the number of months spent at each residence. Discrete binary variables with values 0 or 1 were generated depending on whether the individual was exposed to below (0) or greater than/equal to the median NO_2_ exposure (1) for the sample population within the first year of life.

### Application of information theory metrics to identify interaction effects

The importance of including measures of epistasis (non-additive bio-environmental interactions) in studies of complex traits has risen to the forefront in recent years [17, 19]. The application of information theory metrics to bio-environmental data has been shown to be particularly useful in the identification and characterization of epistatic interactions in studies of complex traits [30]. Information theory defines entropy as a measure of uncertainty, and the information theory metrics of mutual information (MI) and information gain (IG) have been re-discovered by biomedical researchers as entropy-based measures of synergistic (non-additive) interaction effects between biological and environmental variables. For discrete variables, mutual information (MI) quantifies the reduction in uncertainty about the distribution of one variable given an understanding of the other. When applied to case-control studies, MI quantifies the reduction in uncertainty about case/control status given an understanding of single discrete predictor variable. A more intuitive interpretation of MI in the context of case/control studies would be that MI quantifies the independent effect, or main effect, of a single biological or environmental variable on phenotype status.

The measurement of MI can be extended to describe interaction effects by measuring the amount of phenotypic class explained by considering two or more discrete variables, jointly. Information Gain (IG) is the gain in mutual information (MI) on phenotypic class from considering two variables jointly after subtracting the main effect of each individual variable [20, 30]. IG estimates range from − 1 to 1; positive values of IG indicate synergy between the two variables in an interaction model (i.e. a synergistic or epistatic interaction) which negative values of IG indicate redundancy, or correlation, between the two variables in an interaction model. The removal of main effects from the calculation of IG is the main aspect of this information theory metric that makes it an attractive measurement for biomedical researchers interested in discovering interaction effects that are unbiased to the presence of strong main effects.

### Visualization of statistical epistasis networks (ViSEN)

ViSEN is a statistical program that implements the information theory metrics of MI and IG to perform network-based analyses that quantify and visualize pairwise and higher-order epistatic interactions in case control studies. Research suggests that ViSEN is more powerful than standard regression-based methods for detecting additive non-additive interaction effects [19, 26, 33]. ViSEN measures the effects of single explanatory variables on case/control status in terms of MI, and the strength of pairwise interaction effects on case/control status is quantified using IG. ViSEN is particularly suited for the analysis of complex biological traits, as it has extended the calculation of IG to estimate the impact of higher order (more than two predictor variables) interactions on phenotypic class. For higher-order interactions, ViSEN removes not only the main effects (MI) of each independent variable included in the interaction term, but also the effects of each *synergistic* lower order interaction (IG) included in the higher order interaction (Formula 1) [30].

Formula 1. Calculation of Information Gain for Pairwise and Three-variable Interaction Models in ViSEN

$$ {IG}_{pairwise}\left({P}_1;{P}_2;C\right)= MI\left({P}_1;{P}_2;C\right)- MI\left({P}_1:C\right)- MI\left({P}_2;C\right) $$

$$ {IG}_{three- variable}\left({P}_1;{P}_2;{P}_3;C\right)= MI\left({P}_1;{P}_2;{P}_3;C\right)- IG\left({P}_1;{P}_2;C\right)- IG\left({P}_2;{P}_3;C\right)- IG\left({P}_1;{P}_3;C\right) $$

$$ - MI\left({P}_1;C\right)- MI\left({P}_2;C\right)- MI\left({P}_3;C\right) $$

IG: Information Gain; MI: Mutual Information; C: Case/Control Status, P: Predictor Variable.

By subtracting the MI of each single predictor variable from the calculation of IG for pairwise and three-variable models, and also subtracting synergistic pairwise IG from three-variable models, ViSEN is able to capture the “pure” interaction effect only observable when considering all the predictors in the specified interaction model together. It should also be noted that in the case of the higher order IG calculation, by only subtracting synergistic lower-order interaction effects, ViSEN is able to accurately capture higher-order interaction effects even in the case of extreme redundancy, or correlation, between predictor variables [30].

### Application of ViSEN in the current study

Informed by previous studies, we identified age as a potential confounder in our study [13–16, 48]. To address this issue, age needed to be added as a covariate to all interaction analyses to ensure proper interpretation of results. The ViSEN program is currently unable to directly adjust for covariates; therefore, adjustment for age must be performed prior to data analysis. Following a previously published protocol, we applied a local case-control (LCC) subsampling method first presented by Fithian and Hastie (2014) [49, 50].

Briefly, the LLC method involves selecting a subset of individuals from the larger study population that a model containing only the covariates to be adjusted poorly predicts the outcome; data analysis is then restricted to the selected subset of individuals for which the effects of the covariates have been effectively removed. In the context of our study, we used the LLC method to extract a subset of individuals from the 617 SAGE participants with complete phenotype data for which a model containing only age poorly predicted BDR responder status (*n* = 233; females *n* = 97, males *n* = 136) (Table 1, Supplemental Table 1). All downstream interaction analyses were performed in this age-adjusted population. We present participant demographics for the full unadjusted (*n* = 617) and age adjusted (n = 233) in Supplemental Table 1. Participant demographics for sex-stratified analyses are presented in Supplemental Tables 2 and 3.

We calculated pairwise (two-variable) and higher order (three-variable) interaction effects on BDR in the full age- adjusted dataset and in gender-stratified subsets. Permutation (*n* = 1000) was used to generate *p*-values for IG values computed for pairwise and higher order interactions. Permutation datasets were created by randomly shuffling BDR responder status. For each permuted dataset, the IG was recomputed for every pairwise and higher order interaction model to form a null distribution of IG values. *P*-values for IG calculations were generated by comparing the number of permutation-based IG values equal to or larger than the IG value observed in our real dataset. The Bonferroni method was used to control the familywise error rate (FWER) and correct for multiple testing [51, 52]. Bonferroni FWER thresholds of 0.05 and 0.1 were used to generate critical values for significant and suggestive associations, respectively. We performed a total of 28 pairwise tests; the Bonferroni calculated critical value for significant association with BDR for pairwise models was IG permutation p-value < 0.002, and the Bonferroni critical value for suggestive association with BDR was IG permutation p-value < 0.004. We evaluated a total of 56 higher-order (three variable) models; the calculated Bonferroni critical value for significant association with BDR was IG permutation p-value < 0.0009, and the Bonferroni critical value for suggestive association with BDR was IG permutation p-value < 0.002.

### Assessment of ViSEN identified interactions using logistic regression

ViSEN has been shown to be more powerful than standard methods in identifying epistatic interactions. However, since most previous interaction studies employed common statistical methods to identify associations, it is possible that non-additive interactions that significantly impact phenotype were overlooked [20]. Following the standard assumption in biomedical studies of additive main effects and multiplicative interaction effects, we created multiplicative interaction terms to reflect each significant interaction model identified by ViSEN. We then investigated whether any of the newly created interaction terms was significantly associated with BDR using logistic regression. For direct comparison with ViSEN generated results, logistic regression analyses were performed in the same LLC age adjusted dataset that was created for ViSEN analyses. Also, to maintain consistency with ViSEN models, regression models were additionally adjusted for the independent effects of all variants included in the interaction terms as well as lower order interaction models included in the interaction term if applicable. Prior to logistic regression analyses, interaction models were assessed for the presence of multicollinearity and separation using the R packages safeBinaryRegression and mctest, respectively [53, 54]. For instances in which standard logistic regression was inappropriate due to small bin sizes (*n* < 5) (which may have led to separation), an alternative method designed to accommodate these data issues, Firth’s Bias-Reduced Logistic Regression, implemented by the R package logistf, was used instead [55–58]. All regression models assessing pairwise interactions were coded as follows:

$$ \mathrm{BDR}\sim \mathrm{age}+\mathrm{variable}1+\mathrm{variable}2+\mathrm{variable}1\ast \mathrm{variable}2\ \left(\mathrm{pairwise}\ \mathrm{interaction}\ \mathrm{term}\right). $$

Logistic regression models assessing higher-order (three-variable) interaction terms were coded as follows:

$$ \mathrm{BDR}\sim \mathrm{age}+\mathrm{variable}1+\mathrm{variable}2+\mathrm{variable}3+\mathrm{variable}1\ast \mathrm{variable}2+\mathrm{variable}2\ast \mathrm{variable}3+\mathrm{variable}\ 1\ast \mathrm{variable}3+\mathrm{variable}1\ast \mathrm{variable}2\ast \mathrm{variable}3\ \left(\mathrm{three}-\mathrm{variable}\ \mathrm{interaction}\ \mathrm{term}\right). $$

All regression analyses were performed in R [59].

### Post-hoc data visualization and characterization of significantly associated models

For all interaction models that were identified by ViSEN and significantly associated with BDR, after correction for multiple testing, additional visualization and pairwise comparisons were performed to further describe the nature of the relationship between the interaction model and BDR. Boxplots and error plots of the continuous BDR distribution by interaction group membership were generated to visualize the full spread of the data, and the median and IQR, respectively (Supplemental Figure 1, Additional File 5) (Fig. 2). Stacked bar plots describing the percentage of BDR responders and non-responders by interaction group were also generated; bar plots were complemented with *p*-values from the two group test of proportions to describe the significance of differences in proportion of BDR responders between interaction groups. The Bonferroni method was used to correct for multiple testing. All post-hoc data visualization and pairwise testing were performed in R [60].

## Supplementary information

**Additional file 1: Supplemental Table 1**. Participant Demographics in the Full Dataset after Local Case Control Subsampling Adjustment for the Effect of Age. Description of Data: Demographic information for the full dataset before and after adjustment for age using the LCC subsampling method.

**Additional file 2: Supplemental Table 2**. Age Adjusted and Unadjusted Female Subset Demographics. Description of data: Demographic information for female-only analyses in the age adjusted and unadjusted datasets.

**Additional file 3: Supplemental Table 3**. Age Adjusted and Unadjusted Male Subset Demographics. Description of data: Demographic information for male-only analyses in the age adjusted and unadjusted datasets.

**Additional file 4: Supplemental Table 4**. Phenotypic data included for analysis in this study. Description of data: Description and categorization of data included in ViSEN analyses.

**Additional file 5: Supplemental Figure 1**. Full range of Bronchodilator Drug Response Distribution by Pre-natal and Socioeconomic Status (PSE x SES) Interaction Group Membership. Grouped Box Plot Graph of Bronchodilator Drug Response by PSE x SES interaction group membership. Vertical lines denote the group minimum and maximum values, dots represent potential outliers. Boxes represent the interquartile range (IQR) of group data; horizontal lines within boxes indicate the group median. Box color indicates dataset (full, male-only, female-only). Blue horizontal line indicates threshold for clinical response to bronchodilator treatment (∆ FEV_1_ ≥ 12%). ∆ FEV_1_ is the difference in % of Predicted FEV_1_ achieved before and after bronchodilator treatment. ▼ means that the group median < study population median; ▲ means that the group median ≥ study population median. PSE denotes pre-natal smoke exposure, while SES indicates socioeconomic status.

## Data Availability

Biological, environmental and phenotypic data analyzed in the current study are available in the dbGAP repository (study accession number phs00921.v4.p1). Psychosocial data analyzed in the current study (experience of discrimination and socioeconomic status) are not publicly available due to the sensitive nature of the data and privacy concerns for study participants. Psychosocial data is currently stored in the UCSF Box repository and is available from the corresponding author upon reasonable request (https://ucsf.box.com/s/2cx8v52u1ouql02w8io3mhwe2tzell5m).

## Abbreviations

BDR: Bronchodilator drug response
MI: Mutual Information
IG: Information Gain
ViSEN: Visualization of Statistical Epistasis Networks
