# Supplementary material for: An epistatic interaction between pre-natal smoke exposure and socioeconomic status has a significant impact on bronchodilator drug response in African American youth with asthma
Source: BioData Min. 2020 Jul 3;13:7. doi: 10.1186/s13040-020-00218-7 (PMC7333373; doi:10.1186/s13040-020-00218-7)
Supplement: Supplementary file 1 — Additional file 1: Supplemental Table 1. Participant Demographics in the Full Dataset after Local Case Control Subsampling Adjustment for the Effect of Age. Description of Data: Demographic information for the full dataset before and after adjustment for age using the LCC subsampling method. [file 13040_2020_218_MOESM1_ESM.docx]

**Supplemental Table 1. Participant Demographics in the Full Dataset after Local Case Control Subsampling Adjustment for the Effect of Age**

|  | | Raw  Unadjusted Dataset | | | Local Case Control Subsampling  Age Adjusted Dataset | | |
| --- | --- | --- | --- | --- | --- | --- | --- |
|  | | Responders | Non-Responders | P^1^ | Responders | Non-Responders | P^1^ |
| Sample Size, N | | 171 | 446 | --- | 118 | 115 | --- |
| Sex  (% Female) | | 37% | 49% | 0.01 | 42% | 42% | 1.00 |
| Age, yrs.  (Mean, [SE]) | | (14, [0.275]) | (14, [0.169]) | 0.75^2^ | (14, [0.346]) | (14, [0.323]) | 0.37^2^ |
| Body Mass Index | Obese | 63 | 142 | 0.28 | 47 | 39 | 0.42 |
|  | Non-Obese | 108 | 304 |  | 71 | 76 |  |
| Experiences of Discrimination | Yes | 97 | 208 | 0.03 | 65 | 49 | 0.08 |
|  | No | 74 | 238 |  | 53 | 66 |  |
| PSE | Yes | 33 | 86 | 1.00 | 21 | 19 | 0.93 |
|  | No | 138 | 360 |  | 97 | 96 |  |
| SES | > Low | 116 | 290 | 0.57 | 81 | 73 | 0.49 |
|  | Low | 55 | 156 |  | 37 | 42 |  |
| Air Pollution  (NO_2_) | ≥ Median | 91 | 217 | 0.36 | 62 | 54 | 0.47 |
|  | < Median | 80 | 229 |  | 56 | 61 |  |
| Global African Ancestry | ≥ 80% | 116 | 269 | 0.10 | 82 | 67 | 0.10 |
|  | < 80% | 55 | 177 |  | 36 | 48 |  |

The Age Adjusted dataset was created by subsampling the Unadjusted ViSEN Dataset and extracting individuals whose BDR responder status was poorly modeled by age, effectively creating a subset of the data in which the effect of age was removed. All subsequent interaction analyses were carried out in the age adjusted dataset. Summary statistics for all phenotypic data included for analysis in this study are presented above. The Bonferroni method was used to correct for multiple testing (threshold for statistical significance: p-value ≤ 0.006). P-values that remained significant after correction for multiple testing are highlighted in bold. P-values represent the significance of the independent effects, or main effects, of specified variables on BDR responder status. ^1^p-values calculated from χ^2^Chi-squared Test of Independence unless otherwise indicated; ^2^p-values calculated from Wilcoxon Rank Sum test; PSE, Pre-natal Smoke Exposure; SES, Socioeconomic Status.
