# Supplementary material for: An epistatic interaction between pre-natal smoke exposure and socioeconomic status has a significant impact on bronchodilator drug response in African American youth with asthma
Source: BioData Min. 2020 Jul 3;13:7. doi: 10.1186/s13040-020-00218-7 (PMC7333373; doi:10.1186/s13040-020-00218-7)
Supplement: Supplementary file 2 — Additional file 2: Supplemental Table 2. Age Adjusted and Unadjusted Female Subset Demographics. Description of data: Demographic information for female-only analyses in the age adjusted and unadjusted datasets. [file 13040_2020_218_MOESM2_ESM.docx]

|  | | | | **ViSEN** | **Descriptive Statistics** |
| --- | --- | --- | --- | --- | --- |
| Categorical Variable | | BDR  Responders | BDR  Non-Responders | p-value^1^ | p-value^2^ |
| Sample Size, N | | 49 | 48 | --- | --- |
| Age, yrs.  (Mean, [SE]) | | (15, [0.555]) | (14, [0.547]) | 0.67 | 0.37^3^ |
| Body Mass Index | Obese | 17 | 18 | 0.86 | 0.94 |
|  | Non-Obese | 32 | 30 |  |  |
| Experience of Discrimination | Yes | 29 | 20 | 0.01 | 0.13 |
|  | No | 20 | 28 |  |  |
| Prenatal Smoke Exposure | Yes | 13 | 9 | 0.48 | 0.50 |
|  | No | 36 | 39 |  |  |
| Socioeconomic Status | > Low | 36 | 25 | 0.05 | 0.05 |
|  | Low | 13 | 23 |  |  |
| Air Pollution (NO_2_), µg/ppb | ≥ Median | 31 | 23 | 0.17 | 0.19 |
|  | < Median | 18 | 25 |  |  |
| Global African Ancestry | ≥ 80% | 38 | 30 | 0.16 | 0.16 |
|  | < 80% | 11 | 18 |  |  |

**Supplemental Table 2. Female Subset Demographics**

Summary statistics for all phenotypic data included for analysis in this study are presented above. The Bonferroni method was used to correct for multiple testing (threshold for statistical significance: p-value ≤ 0.006). P-values that remained significant after correction for multiple testing are highlighted in bold. P-values represent the significance of the independent effects, or main effects, of specified variables on BDR responder status. ^1^p-values calculated from ViSEN’s Mutual Information (MI) Test. MI is a metric that quantifies the reduction in uncertainty about the distribution of one variable given an understanding of the other; ^2^p-values calculated from χ^2^ Test of Independence unless otherwise indicated; ^3^p-values calculated from Wilcoxon Rank Sum test.
