# Supplementary material for: An epistatic interaction between pre-natal smoke exposure and socioeconomic status has a significant impact on bronchodilator drug response in African American youth with asthma
Source: BioData Min. 2020 Jul 3;13:7. doi: 10.1186/s13040-020-00218-7 (PMC7333373; doi:10.1186/s13040-020-00218-7)
Supplement: Supplementary file 3 — Additional file 3: Supplemental Table 3. Age Adjusted and Unadjusted Male Subset Demographics. Description of data: Demographic information for male-only analyses in the age adjusted and unadjusted datasets. [file 13040_2020_218_MOESM3_ESM.docx]

|  | | | | **ViSEN** | **Descriptive Statistics** |
| --- | --- | --- | --- | --- | --- |
| Categorical Variable | | BDR  Responders | BDR  Non-Responders | p-value^1^ | p-value^2^ |
| Sample Size, N | | 69 | 67 | --- | --- |
| Age, yrs.  (Mean, [SE]) | | (13, [0.425]) | (13, [0.392]) | 0.87 | 0.46^3^ |
| Body Mass Index | Obese | 30 | 21 | 0.17 | 0.20 |
|  | Non-Obese | 39 | 46 |  |  |
| Experience of Discrimination | Yes | 36 | 29 | 0.33 | 0.39 |
|  | No | 33 | 38 |  |  |
| Prenatal Smoke Exposure | Yes | 8 | 10 | 0.65 | 0.75 |
|  | No | 61 | 57 |  |  |
| Socioeconomic Status | > Low | 45 | 48 | 0.47 | 0.50 |
|  | Low | 24 | 19 |  |  |
| Air Pollution (NO_2_), µg/ppb | ≥ Median | 31 | 31 | 1.00 | 1.00 |
|  | < Median | 38 | 36 |  |  |
| Global African Ancestry | ≥ 80% | 44 | 37 | 0.38 | 0.40 |
|  | < 80% | 25 | 30 |  |  |

**Supplemental Table 3. Male Subset Demographics**

Summary statistics for all phenotypic data included for analysis in this study are presented above. The Bonferroni method was used to correct for multiple testing (threshold for statistical significance: p-value ≤ 0.006). P-values that remained significant after correction for multiple testing are highlighted in bold. P-values represent the significance of the independent effects, or main effects, of specified variables on BDR responder status. ^1^p-values calculated from ViSEN’s Mutual Information (MI) Test. MI is a metric that quantifies the reduction in uncertainty about the distribution of one variable given an understanding of the other; ^2^p-values calculated from χ^2^ Test of Independence unless otherwise indicated; ^3^p-values calculated from Wilcoxon Rank Sum test
