# Supplementary figures and images for: An epistatic interaction between pre-natal smoke exposure and socioeconomic status has a significant impact on bronchodilator drug response in African American youth with asthma
Source: BioData Min. 2020 Jul 3;13:7. doi: 10.1186/s13040-020-00218-7 (PMC7333373; doi:10.1186/s13040-020-00218-7)

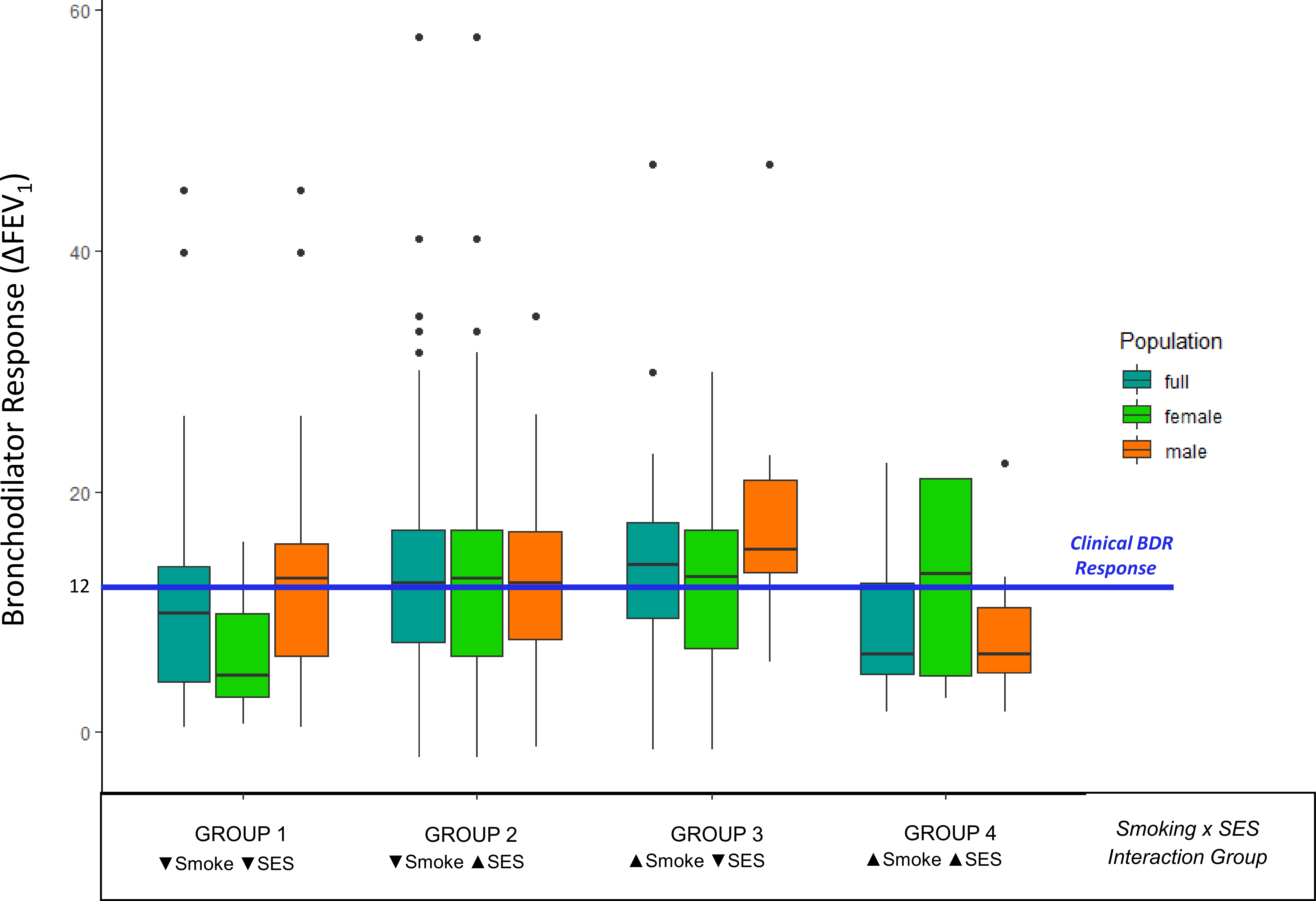

Supplement: Supplementary file 5 — Additional file 5: Supplemental Figure 1. Full range of Bronchodilator Drug Response Distribution by Pre-natal and Socioeconomic Status (PSE x SES) Interaction Group Membership. Grouped Box Plot Graph of Bronchodilator Drug Response by PSE x SES interaction group membership. Vertical lines denote the group minimum and maximum values, dots represent potential outliers. Boxes represent the interquartile range (IQR) of group data; horizontal lines within boxes indicate the group median. Box color indicates dataset (full, male-only, female-only). Blue horizontal line indicates threshold for clinical response to bronchodilator treatment (∆ FEV1 ≥ 12%). ∆ FEV1 is the difference in % of Predicted FEV1 achieved before and after bronchodilator treatment. ▼ means that the group median < study population median; ▲ means that the group median ≥ study population median. PSE denotes pre-natal smoke exposure, while SES indicates socioeconomic status. [file 13040_2020_218_MOESM5_ESM.tif]
